# Mapping Global Scientific Production on Human Immunodeficiency Virus and Monkeypox: A Scientometric Study

**DOI:** 10.1002/hsr2.70469

**Published:** 2025-03-09

**Authors:** Frank Mayta‐Tovalino, Carlos Quispe‐Vicuña, Cesar Mauricio‐Vilchez, Diego Galarza‐Valencia, Fran Espinoza‐Carhuancho

**Affiliations:** ^1^ Vicerrectorado de Investigación, Universidad San Ignacio de Loyola Lima Peru; ^2^ Red de Eficacia Clínica y Sanitaria (REDECS) Lima Peru; ^3^ Academic Department, Faculty of Medical Technology Universidad Nacional Federico Villarreal Lima Peru; ^4^ Unidad de Investigación, Innovación y Emprendimiento Universidad Nacional Federico Villarreal Lima Peru; ^5^ Grupo de Bibliometría, Evaluación de evidencia y Revisiones Sistemáticas (BEERS), Human Medicine Career Universidad Cientifica del Sur Lima Peru

**Keywords:** bibliometrics, global public health, HIV, Mpox

## Abstract

**Background and Aim:**

HIV attacks the immune system, leading to AIDS if untreated. Mpox, a zoonotic disease like smallpox, is less severe but poses higher risks for immuno‐compromised individuals, especially those with HIV. Effective prevention and treatment are crucial. This study aims to assess the global academic output on Monkeypox (MPVX) and HIV during 2018–2023.

**Methods:**

This descriptive, bibliometric study reviewed the published literature on “monkeypox” and “HIV” during the period 2018–2023. The search was conducted on February 11, 2024, using a systematic formula. We identified 366 potential articles, including various types of papers. When exporting metadata to SciVal for the period 2018–2023, 8 metadata could not be exported. The remaining data were analyzed using SciVal and Bibliometrix in R Studio, providing an overview of research productivity, collaborations, and citation impact.

**Results:**

During the period 2018–2023, 366 papers on “monkeypox” and “HIV” were published in 183 different sources, with an annual growth rate of 208.18%. These papers had an average of 14.78 citations per paper. An additional 2522 keywords and 638 author keywords were identified. The “*Journal of Medical Virology*” led the academic output with 20 papers, followed by “*The Lancet Infectious Diseases*” with 11 papers. The “*Institut national de la santé et de la recherche médicale*” in France led the academic output. These data are specific to papers dealing with “monkeypox” and “HIV.”

**Conclusion:**

Research on Monkeypox and HIV has grown significantly during the period studied, with remarkable international collaboration. These findings underscore the importance and global impact of Monkeypox and HIV research.

## 1 Introduction

Monkeypox (MPXV) is a viral zoonosis, first identified in 1970 whose symptoms are similar, but less severe, to those caused by smallpox and its development is usually endemic to areas such as Central and West Africa [1]. However, in 2003 it had a first outbreak in a nonendemic area such as the United States; in 2018 in Israel, in the United Kingdom in 2019 [2].

Recently, there has been an abrupt increase in the number of outbreaks in the United States spreading to the rest of the world reaching more than 66,000 cases in more than 100 nonendemic countries [1], representing approximately 4% of all global infections especially in male patients who have sex with men (MSM) and being declared by WHO as a public health emergency on July 23, 2022 [3]. This spread is also conditioned by various risk factors such as the immunological status of the patient.

Approximately a total of 30.18% of patients with MPXV are patients with human immunodeficiency virus (HIV) [3]. In cases of MPXV patients with uncontrolled advanced HIV infection, there is an inadequate immune response and consequently a weak prognosis, a longer period of illness and a delayed cure [4]; although the association between the two diseases is not entirely clear, the cause may lie in the similar modes of transmission (sexual route) [5].

Co‐infection of these two viruses can be particularly dangerous because it can exacerbate the symptoms of both diseases [6]. This is why it is necessary to systematically compare HIV and MPXV data to get a clear picture of the status of coinfection in all regions of the world because the mode of transmission of the two diseases may vary in different countries. To achieve this, bibliometric studies are a good option to perform a quantitative analysis of the current medical literature on a given topic [7].

The results of this study will serve as a basis for a clear and current overview of MPVX coinfection in patients with HIV and vice versa, emphasizing important topics to promote the improvement of medical practice and public health policies. Despite the great extension and impact of MPVX in the world and in the research area, bibliometric analyses have only focused on the disease in general [8] or evaluating the various therapies and vaccines it presents [9], without analyzing this coinfection.

Thus, the aim of this study was to conduct a scientometric study of the world literature on HIV and MPXV coinfection.

## 2 Methods

### 2.1 Study Design

The study had a descriptive design with a scientometric approach. A review of the literature published on “monkeypox” and “HIV” during the period 2018–2023 was conducted using Scopus.

### 2.2 Search Process

The search was conducted on February 11, 2024 using the following formula: TITLE‐ABS (“monkeypox” OR “mpox” OR “simianpox” OR “primatepox” OR “apepox” OR “macaquepox” OR “saimiripox” OR “baboonpox” OR “chimpox” OR “gorillapox”) AND TITLE‐ABS (“HIV” OR “human immunodeficiency virus” OR “acquired immune deficiency syndrome” OR “hiv‐positive” OR “retrovirus” OR “human immunodeficiency syndrome virus” OR “acquired immunodeficiency syndrome virus”) AND PUBYEAR > 2017 AND PUBYEAR < 2024.

### 2.3 Article Selection

We found 366 potential articles on “monkeypox” and “HIV.” These include 271 articles, 50 reviews, 22 letters, 12 notes, 4 editorials, 3 errata, 3 book chapters, and 1 conference article.

### 2.4 Criteria Selection

The inclusion criteria for this study were articles published between 2018 and 2023 that included the terms “monkeypox” in the title or abstract, focusing on both “monkeypox” and “HIV.” The exclusion criteria involved articles that did not meet the search terms, were published outside the specified timeframe (before 2018 or after 2023), did not focus on both “monkeypox” and “HIV,” or had metadata that could not be exported to SciVal for analysis.

### 2.5 Data Export

When exporting metadata to SciVal for 2018–2023, 8 metadata could not be exported. The remaining data were used for bibliometric analysis. Data analysis was performed using SciVal and Bibliometrix in R Studio. In SciVal, metadata of selected papers were imported for detailed bibliometric analysis. SciVal provided an overview of research productivity, collaborations, and citation impact. On the other hand, Bibliometrix, an R package designed for bibliometric and scientometric analysis, was used for additional data processing. With Bibliometrix, a keyword co‐occurrence analysis was performed to identify the main research areas. It was also used to visualize the collaboration network between authors and countries.

### 2.6 Data Analysis

Data were analyzed using standard bibliometric techniques to identify trends in scholarly output, collaborations, author productivity, and keyword distribution. Academic output, measured by number of publications, CiteScore 2022, Field Weighted Citation Impact, citations per publication, and views per publication were used.

## 3 Results

In the period 2018–2023, 366 papers were published on “monkeypox” and “HIV” in 183 different sources, with an annual growth rate of 208.18%. These papers have an average of 14.78 citations per paper. An additional 2522 keywords and 638 author keywords were identified. The papers were written by 3362 authors, of which 19 wrote single‐authored papers. The average number of coauthors per paper is 10.9, with 19.4% international coauthor ships. Paper types include articles (271), book chapters (3), conference papers (1), editorials (4), errata (3), letters (22), notes (12), and reviews (50) (Table 1).

**Table 1:** Descriptive characteristics of scientific production.

Description	Results
Perior	2018:2023
Sources	183
Documents	366
Annual growth (%)	208.18
Document average age	1.28
Average citations per doc	14.78
References	9001
Keywords plus	2522
Author's keywords	638
Authors	3362
Authors of single‐authored docs	19
Single‐authored docs	22
Co‐authors per doc	10.9
International co‐authorships %	19.4
Article	271
Book chapter	3
Conference paper	1
Editorial	4
Erratum	3
Letter	22
Note	12
Review	50

The “Journal of Medical Virology” led the academic output with 20 papers, followed by “The Lancet Infectious Diseases” with 11 papers and “International Journal of STD and AIDS” and “Travel Medicine and Infectious Disease” with 10 papers each. “The Lancet” has the highest CiteScore in 2022 with 133.2 and also led in citations per publication with 72.7. However, “The Lancet Infectious Diseases” had the highest field‐weighted citation impact with 34.3. It is important to note that these data are specific to papers dealing with “monkeypox” and “HIV” (Table 2).

**Table 2:** Top‐10 scientific journals.

Source	Scholarly output	CiteScore 2022	Field‐weighted citation impact	Citations per publication
*Journal of Medical Virology*	20	23.4	6.28	9.6
*The Lancet Infectious Diseases*	11	55.6	34.3	49.8
*International Journal of STD and AIDS*	10	2.3	5.28	10.2
*Travel Medicine and Infectious Disease*	10	17.1	3.43	11.8
*AIDS*	8	6.3	2.43	2.8
*AIDS Research and Human Retroviruses*	7	3.2	0.66	0.7
*The Lancet*	7	133.2	45.91	72.7
*Vaccines*	7	7	4.25	10
*Clinical Microbiology and Infection*	6	21.5	14.75	17.7
*Infectious Disease Reports*	6	3.2	1.74	5.7

The “Institut national de la santé et de la recherche médicale” in France led the academic production with 15 papers, followed by the “Centro de Investigación Biomédica en Red” and the “Instituto de Salud Carlos III” in Spain with 14 papers each. The Université Paris Cité in France produced 13 papers. In the United States, the University of Miami and Emory University published 12 and 11 papers respectively. The “Assistance publique ‐ Hôpitaux de Paris” in France and the “CNRS” in France, together with the “Centers for Disease Control and Prevention” in the United States and the “IRCCS Istituto per le Malattie Infettive Lazzaro Spallanzani ‐ Roma” in Italy, produced 11 and 10 papers respectively (Table 3).

**Table 3:** Top‐10 institutions.

Institution	Country	Scholarly output	Views per publication	Field‐weighted citation impact	Citations per publication
Institut national de la santé et de la recherche médicale	France	15	13.9	27.6	34.7
Centro de Investigación Biomédica en Red	Spain	14	16.3	44.55	89.2
Instituto de Salud Carlos III	Spain	14	16.3	44.55	89.2
Université Paris Cité	France	13	17.4	65.77	111.2
University of Miami	United States	12	5.4	3.63	10.7
Assistance publique—Hôpitaux de Paris	France	11	16.3	78.69	128.5
CNRS	France	11	17.5	30.21	39.3
Emory University	United States	11	12.6	17.56	30.5
Centers for Disease Control and Prevention	United States	10	6.9	12.45	10.4
IRCCS Istituto per le Malattie Infettive Lazzaro Spallanzani—Roma	Italy	10	18.1	58.55	116.7

**Figure 1:**
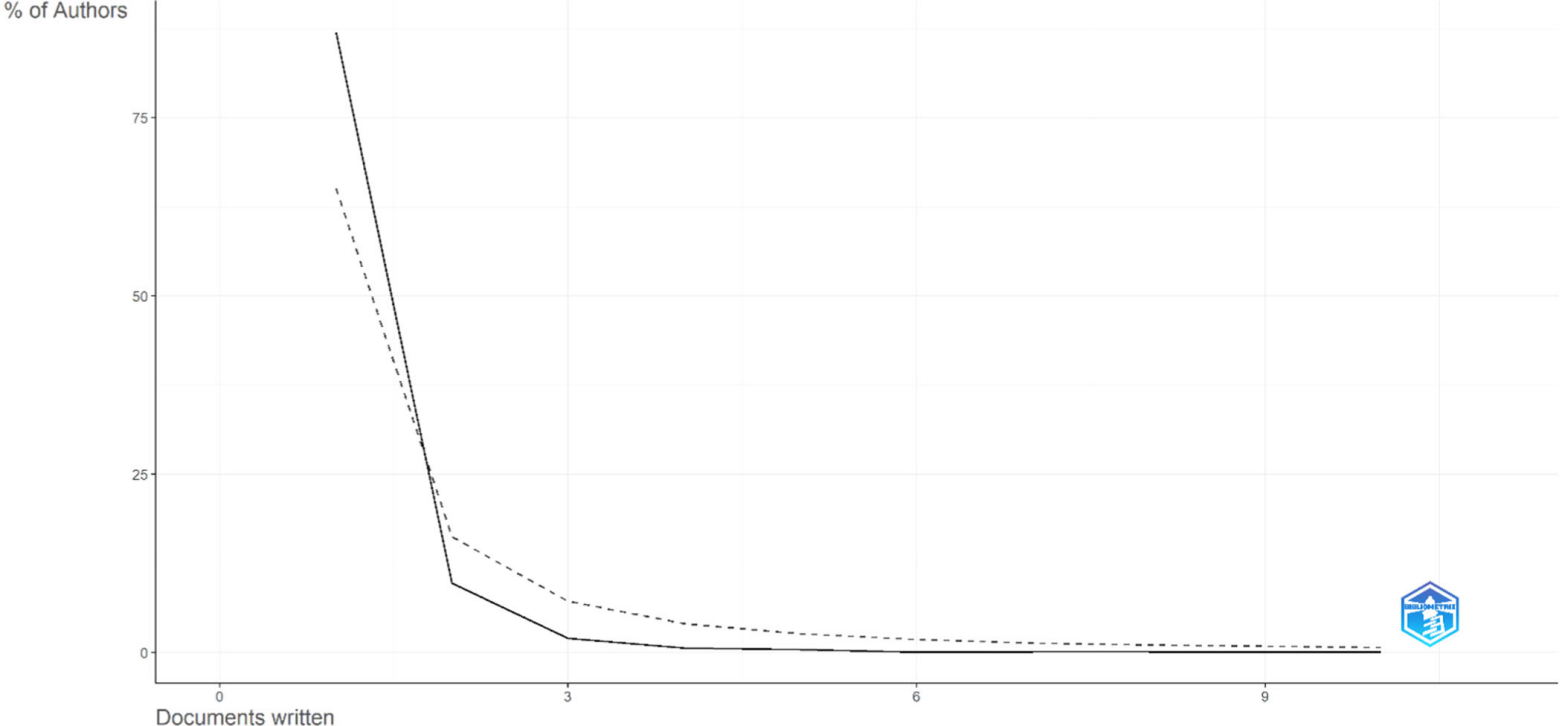
Most relevant authors.

Most authors (2925, representing 87% of the total) wrote only one paper. A smaller number of authors (328, or 9.8% of the total) wrote two papers. As the number of papers written per author increases, the proportion of authors decreases. For example, only 66 authors (2% of the total) wrote three papers, and only 21 authors (0.6% of the total) wrote four papers. Very few authors wrote more than five papers. These data reflect the typical distribution of academic productivity, where most authors publish few papers and only a few publish many (Figure 1).

**Figure 2:**
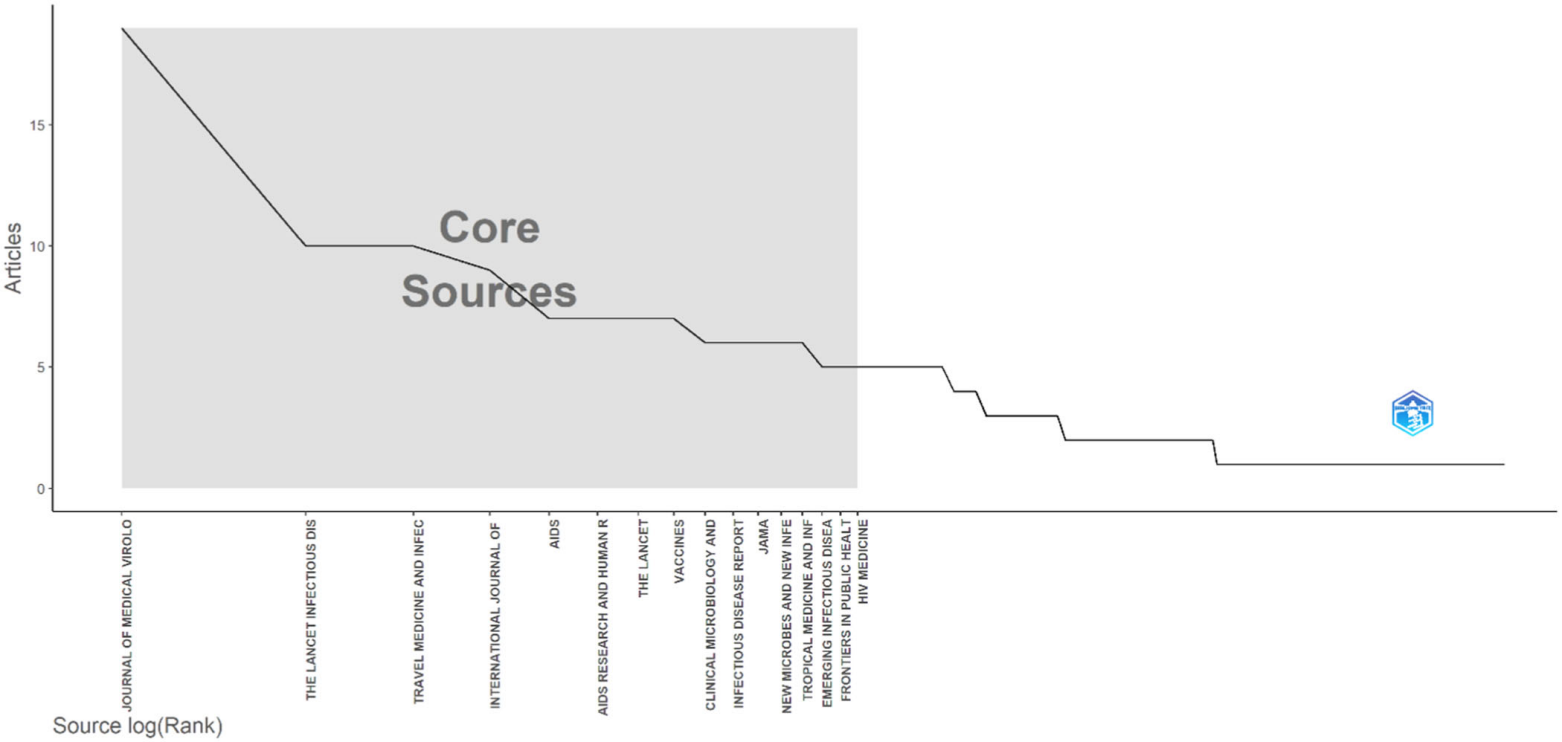
Core sources.

According to Bradford's law, monkeypox and HIV publications were distributed into three zones. Zone 1, which includes the most productive journals, comprised 15 journals, from the Journal of Medical Virology to Frontiers in Public Health. Zone 2, with moderate productivity, included 46 journals, from HIV Medicine to Sexual Health. Zone 3, which includes journals with only one publication, started with “Abdominal Radiology” and continued with journals such as “Acta Oto‐Laryngologica,” “Advanced Emergency Nursing Journal,” and “Advances in Experimental Medicine and Biology” (Figure 2).

**Figure 3:**
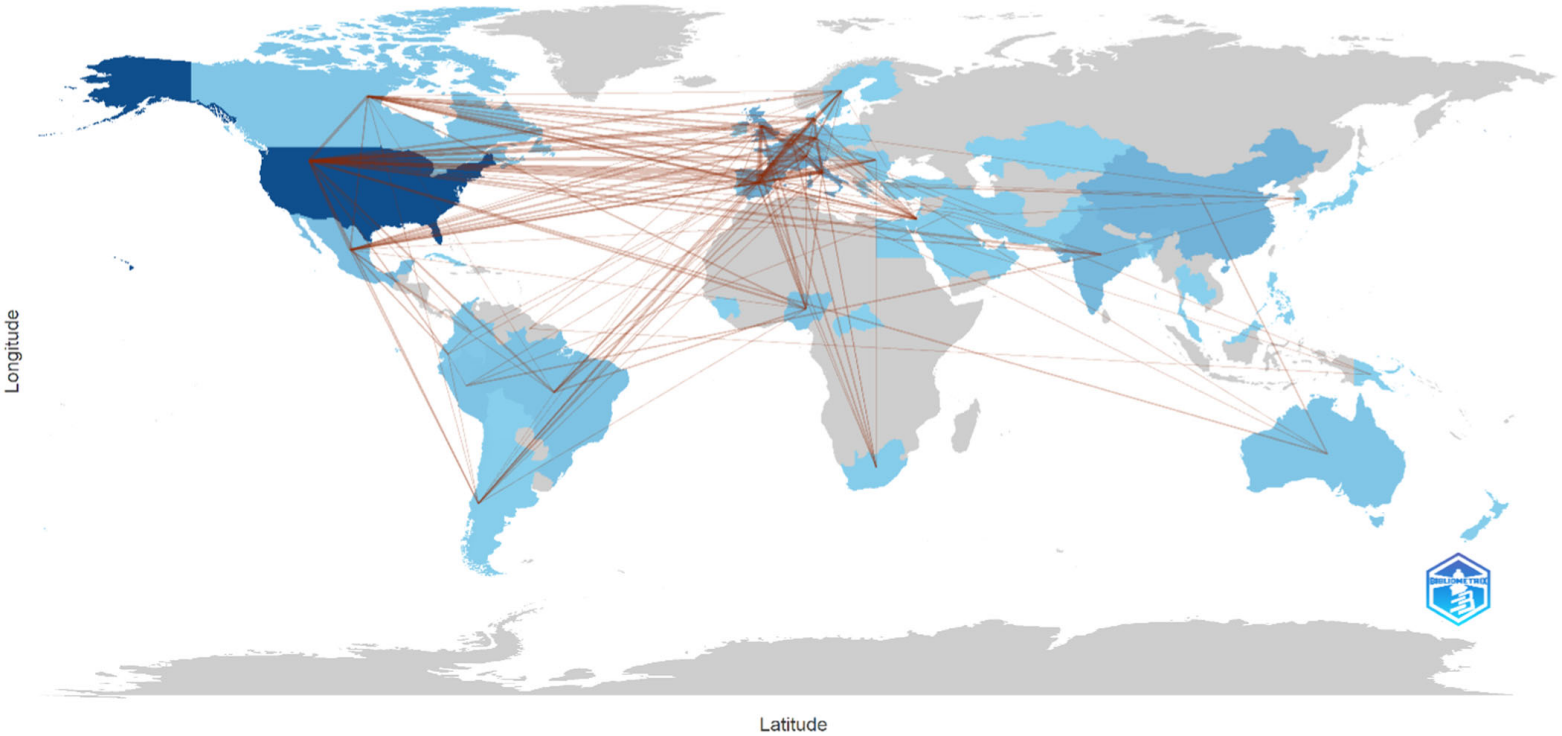
Country collaboration map.

The map of collaboration between countries shows several interactions. France has the most collaborations, with six with Germany, five with Canada and Switzerland, and three with Belgium, Brazil, Denmark, Israel, Mexico, Nigeria, Portugal, Romania and Sweden. Germany also shows a strong network of collaborations, with four with Canada, Denmark, Mexico and Sweden. Canada and Denmark have three collaborations with Belgium. Brazil has three collaborations with Nigeria. These data highlight the global connections and collaboration between these countries (Figure 3).

## 4 Discussion

In the present study, 366 documents were found, most of which were articles and reviews with an annual growth rate of 208.18% and an average of 14.78 citations per article. In addition, 19.4% of international collaboration was found, especially from European countries such as France or Germany.

Co‐infection with HIV and Mpox can significantly worsen the progression of both diseases, leading to more severe clinical outcomes. Individuals with advanced HIV and low CD4 cell counts are especially susceptible to severe Mpox complications, such as necrotizing skin lesions, pulmonary involvement, and secondary infections. The co‐infection can trigger a state of CD4+ T cell activation, which facilitates HIV infection. Overall, HIV co‐infection tends to exacerbate the prognosis of Mpox, increasing both mortality and the severity of complications [10, 11, 12].

The Lancet was the journal with the highest CiteScore 2022 and the highest impact (72.7 citations per paper) with only 7 publications. This journal has already reported in previous years leading the global scientific production in the topic of infectious diseases [13] as well as periodically reporting the impact of pandemics on global public health as was COVID‐19 in previous years [14], which would explain its higher impact in the topic of MPXV and HIV. The article with the highest citation (359) in this journal was that of Tarín‐Vicente et al. [15], which was a prospective multicenter cohort in Spain that sought to describe the clinical and virological characteristics of 181 patients diagnosed with MPXV, finding that 40% were co‐infected with HIV.

The Assistance publique—Hôpitaux de Paris was the institute with the highest reported impact with 128.5 publications per paper. This is in line with the world literature where this institution is identified as the largest university hospital in Europe and one of the major references of French research since it encompasses about 40% of French biomedical research, especially during the last COVID‐19 pandemic [16]. Among its most cited publications is Mitjà et al. [10] which was a case series describing 382 cases from 19 countries with a diagnosis of MPXV of which 91% had HIV comorbidity.

Eighty‐seven percent of the authors wrote only one paper and similarly as the number of papers written per author increases, the proportion of authors decreases. This reflects a typical picture of academic productivity, with most authors publishing only one paper. It has been reported that during the surge of publications on MPXV during its pandemic, the majority (5800 authors) published only one paper and this picture was quite like that of the previous COVID‐19 pandemic [17]. Despite this, international collaboration in publications on MPXV and HIV was reported, which is in keeping with the increase in cases and the global emergency that both viruses are causing worldwide.

The higher concentration of both publications and collaborative networks in European countries responds to the very extension of MPXV in the world. The recent outbreak of MPXV in several countries and in nonendemic countries was found to have a higher prevalence of HIV coinfection (42.05% and 42.60%, respectively) [11] and up to a 100‐fold difference in cases compared with HIV prevalence in the global population [12]. This could be explained by the fact that nonendemic European and American countries have a higher proportion of the population of MSM, bisexuals and homosexuals [18], which are HIV risk groups.

These results highlight the importance of constant vigilance in immunosuppressed patients such as HIV‐positive patients by prioritizing vaccination against MPVX in this population and, similarly, HIV screening in patients diagnosed with MPVX [5]. It is also hoped that our results will lay the groundwork for future research and improved public policy on health care, emphasizing appropriate clinical guidance for MPVX.

This study has several limitations. First, the search was limited to the Scopus database only, which may have excluded relevant articles from other databases. Second, the classification of the publications (article, review, letter, etc.) may not fully reflect the content of the articles. Third, the study was limited to articles published between 2017 and 2024, which excluded case reports and research before the MPVX pandemic. However, this study also has some strengths. First, a systematic analysis of the Scopus database was performed, which covers many journals and publications that have undergone a strict review process to ensure high quality and relevance to the global literature compared with other databases [19]. Second, the classification of various types of publications (articles, reviews, letters, book chapters, etc.) allows a more complete and diverse view of the typical study. Finally, considering studies published in the last few years allows us to address a current picture of the reality on the conflation of MPVX and HIV.

## 5 Conclusions

The bibliometric study of global academic output on Monkeypox and HIV during 2018–2023 revealed significant growth in research, with 366 papers published in 183 different sources. International collaboration was notable, with 19.4% international co‐authorship. Papers were distributed in a variety of types, including articles, book chapters, and reviews. Publications are led by the *Journal of Medical Virology* and *The Lancet Infectious Diseases*. Academic productivity was led by the Institut national de la santé et de la recherche médicale in France and the Centro de Investigación Biomédica en Red in Spain. Most authors published few papers, reflecting the typical distribution of academic productivity. Collaborations between countries are global, highlighting the connections and collaborations between these countries.

## Author Contributions

**Frank Mayta‐Tovalino:** conceptualization, investigation, writing – review and editing, software, data curation, supervision. **Carlos Quispe‐Vicuña:** investigation, conceptualization, writing – original draft. **Cesar Mauricio‐Vilchez:** conceptualization, methodology, investigation, writing – original draft. **Diego Galarza‐Valencia:** conceptualization, investigation, methodology, writing – original draft. **Fran Espinoza‐Carhuancho:** investigation, writing – original draft, conceptualization, methodology.

## Conflicts of Interest

The authors declare no conflicts of interest.

## Transparency Statement

The lead author Frank Mayta‐Tovalino affirms that this manuscript is an honest, accurate, and transparent account of the study being reported; that no important aspects of the study have been omitted; and that any discrepancies from the study as planned (and, if relevant, registered) have been explained.

## Data Availability

The data that support the findings of this study are available from the corresponding author upon reasonable request.
